# Correlation between input on public health services and work motivation among primary health workers in China

**DOI:** 10.1186/s12875-023-01986-x

**Published:** 2023-01-27

**Authors:** Jia Li, Yahang Yu, Luping Hei, Beibei Yuan

**Affiliations:** 1 China Center for Health Development Studies, Peking University, Beijing, China; 2 Department of Health Policy and Management, School of Public Health, Peking University, Beijing, China

**Keywords:** Public health services, Intrinsic work motivation, Primary health workers, China

## Abstract

**Background:**

The integration of public health services into primary health care has been advocated and practiced worldwide for better management of preventable diseases. Health policy makers in China have started the reforms to better integrate public health services and clinical services, but public health services in China still remained neglected in primary health system. This study aimed to explore the input of Chinese primary health workers on delivering public health services and its association with their intrinsic working motivation in China.

**Methods:**

Data were collected from a cross-sectional survey conducted in 2019. Participants in this survey included 803 primary health workers in 75 primary health institutions in China. Questions about the input on clinical and public health services delivery and intrinsic working motivation were asked. A multiple linear regression model was adopted to investigate the correlation between intrinsic working motivation and the time input on public health service. The robustness of this model was checked with a generalized linear model.

**Results:**

Intrinsic motivation was found to have negative association with health workers’ input on public health (β: -1.01, *p* < 0.05), with the robustness checked with a generalized linear model. The significance of this association differed in the group of urban community health centers and rural township health center. Other factors that had significant relationship with the input on public health services include the being nurses instead of doctors (*p* < 0.01), being a member of family doctor team (*p* < 0.01), recognition on relative importance of clinical services (*p* < 0.01), and perception on better exterior support (*p* < 0.01).

**Conclusion:**

With higher intrinsic working motivation, primary health workers tended to spend less time on public health services. It reflected that doctors and nurses in primary healthcare institutions still perceived clinical treatment services as their main work responsibility and source of career recognition. Organizational level supports and system level policies should guide the primary health workers to increase their awareness on the importance of public health services and to cultivate their internal interests on public health services, in order to ensure sustainable input and performance improvement on public health services in primary health system in China.

## Background

As the prevalence of chronic diseases increased year by year, the integration of preventive services or health management services (such as health education and regular follow-up) into primary care became essential in chronic disease control and management. Primary care refers to “the provision of integrated, accessible health care services by clinicians who are accountable for addressing a large majority of personal health care needs, developing a sustained partnership with patients, and practicing in the context of family and community” [1]. However, previous primary care in both developed and developing countries tended to attach more attention on clinical or curative health care targeted at individual patient [2], and neglected population health and public health services. The challenges of aging population and rising burden of chronic diseases are driving primary care to transform from passively diagnosing and treating diseases to proactively preventing diseases and undertaking health management. For chronic diseases, controlling risk factors and conducting health management during the whole course of diseases are of the same value as the clinical treatment services for diseases.

Public health is defined as “the science and art of preventing disease, prolonging life and promoting health through the organized efforts of society” [3]. According to American Public Health Functions Steering Committee, essential public health services usually include ten items such as monitoring health status to identify and solve community health problems, informing, educating, and empowering people about health issues, mobilizing community partnerships and action, and so on [4]. The integration of public health into primary care has been advocated in different health system settings, as it was believed that integration care enabled the delivery of more effective and comprehensive services, and help with better management of diseases in communities [5].

At the beginning, primary health care system in China was designed to undertake both public health services and curative services. However, most primary health institutions (PHIs) in China set separated departments for the two kinds of services, and public health services and curative services are reimbursed by different financing system [6]. PHIs held strong motivation in providing more curative services other than public health services, which was attributed to the financing structure of PHIs: the subsides on public health services are relatively low; meanwhile, the revenues could be obtained from curative services which were paid from out-of-pocket payment of patients or social insurance reimbursement through fee-for-service method. At the same time, the awareness and recognition on the importance of preventing diseases in population is not sufficient in doctors and nurses of PHIs [7]. As the consequence, doctors of PHIs had stronger willingness in delivering more curative services, although they had to work on public health services under supervision pressure from administration department.

In recent years, health system reforms in China have begun to emphasize the integration of disease prevention and management into routine disease treatment services in primary care in several official documents [8, 9] and have started piloting different integration models in several provinces [10]. Nonetheless, so far, the implementation of polices showed limited progress and effectiveness. Several studies have analyzed the barriers preventing integration of two kinds of services in primary health system in China. The majority focused on the system or organizational level arrangements, including the segmented financing sources and the separated administration departments for two kinds of services. Few studies analyzed the barriers on the individual level of health workers, espeically the working motivation of health workers which drives the direction and effort level of health workers’ behaviours.

Work motivation was defined as “an energy or a force that arises from within an individual, or from the environment, to take on work-related behavior, and to shape this behavior’s form, intensity, and duration”[11] Higher levels of work motivation have been proven significantly correlated with higher levels of organizational performance [12]. The casual relationship between work motivation and job performance was studied and explained with many different theories, including the self-determined theory [13], achievement goal theory [14], and job characteristic model [15]. In various professionals, including those of the health sector, motivation was confirmed as an essential part in achieving and maintaining high-quality performance of health workers, especially in low-resource settings [16]. Health service delivery process, service quality, efficiency and equity were all related to the motivation of health workers [17]. Studies on work motivation also focused on its correlation with feelings in work: some studies exploring influence of motivation investigated its close relationship with job burnout [18] and turnover intention [19] among health workers; it has also been proposed that work motivation can affect job satisfaction, hence influencing job performance [20, 21]. A study in Nigeria [22] found better performance of village health workers on public health services, mainly in the area of the maternal and child health care, was correlated to higher work motivation through feelings of confidence and sense of achievements.

With greater emphasis placed on external regulators of motivation such as economic incentives and environmental support, the role of intrinsic motivation among health workers remained an area with scarce notice. Intrinsic motivation was described the innate propensity to pursue interesting tasks that challenge one’s skills and foster growth [23]. Karatepe and Tekinkus [24] demonstrated that high levels of intrinsic motivation resulted in high levels of job performance, job satisfaction, and affective commitment to the organization. In health sector, there were evidences [25–27] suggesting that intrinsic motivation and work enthusiasm positively influenced healthcare professionals’ performance in health service delivery. In theory, as one important determinant of attitudes in work, intrinsic motivation would be a potential influencing factor of the successful integration of public health into primary care. However, there are few studies exploring the relationship between work motivation and preference or choice of health workers between public health services and medical services. Only Valaitis et al. [28] conducted an interpretative study to explore stakeholders’ perceptions on successful primary care and public health collaboration, and found that the innate propensity within a person such as personal values, beliefs, and attitudes were influencing factors in building and sustaining primary care and public health collaboration.

There is lack of evidence on the determinants of primary health workers’ choice and behaviors in integration of public health services into their medical works. In addition, previous studies focusing on factors that may facilitate the delivery of public health services in Chinese primary care situations mainly investigated external administrative, financial and technical support factors [29], lacking attentions to the influence of individual work motivation. To address these gaps, and to complement more evidence on the individual level determinants of integration care in primary healthcare settings, this study used empirical data from a cross-sectional survey to display Chinese primary health workers’ input in delivering public health services, explore its association with health workers’ intrinsic motivation, and provide relevant suggestions to encourage better integration of public health services into primary care based on empirical findings.

## Methods

### Study settings and participants

**Fig. 1:**
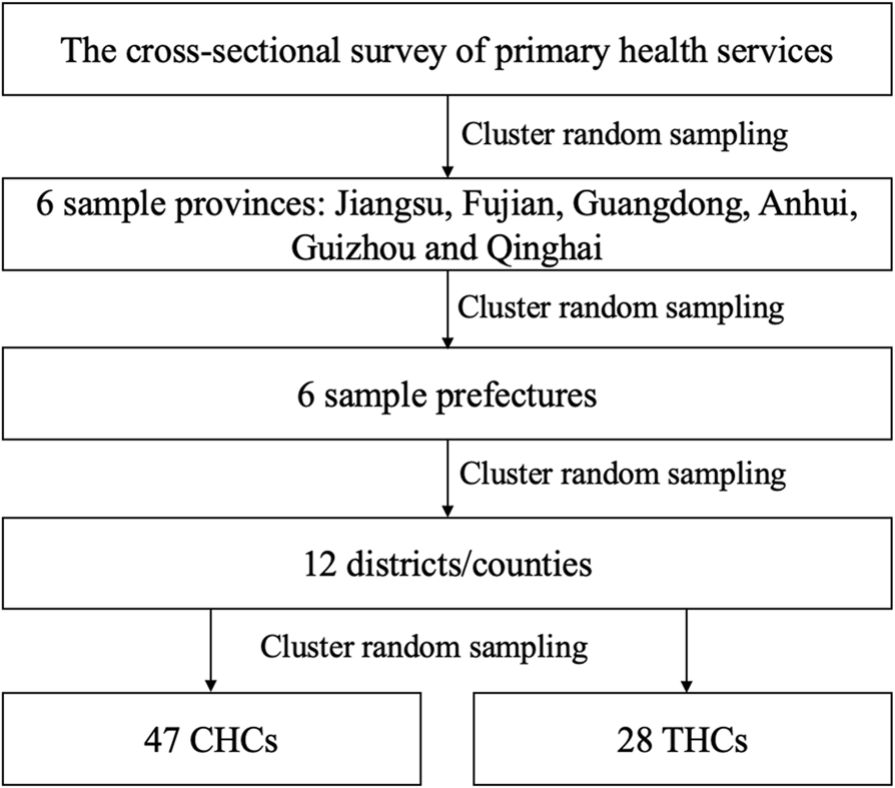
The sampling process and method

This study utilized data collected in a cross-sectional survey of primary health facilities covering six provinces in China from April to October, 2019. The survey adopted the multi-stage cluster random sampling method. The whole sampling process was described in Fig. 1. The socioeconomic development varied across China, on most occasions, the eastern region represented the most developed area in China since it covered the highest proportion of GDP, and the central region and western region ranked second and the least. In addition, health system development also differentiates among the three regions. According to the China Health Statistics Yearbook 2020, the eastern region accounted for the largest proportions of the number of PHIs and the number of primary health professionals in 2019, which were 37.8% and 42.2%, respectively, while the western region accounted for the lowest of both indicators, which were 31.0% and 28.7%. Therefore, Jiangsu, Fujian, and Guangdong provinces of the eastern region, Anhui province of the central region, and Guizhou and Qinghai provinces of the western region were selected as sample provinces representing different economic levels and health system development of China.

A sample prefecture was then selected for each province. We randomly selected two districts/counties in each prefecture. Twelve PHIs in the two selected districts/counties including 6 community healthcare centers (CHCs) in urban areas and 6 township healthcare centers (THCs) in rural areas were selected using cluster random sampling method. If there were no rural area under the jurisdiction of the sample city, the 12 PHIs were all selected from CHCs. Totally 75 PHIs including 47 CHCs in the urban areas and 28 THCs in the rural areas were investigated in this survey. All the on-duty health workers including 437 doctors and 366 nurses on the investigation day in the 75 PHIs were asked to finish a self-administrated questionnaire, covering a series of questions concerning the basic sociodemographic information, their input on curative and public health services delivery, and a working motivation scale. The final samples were made up of 803 health workers comprising 60.2% of them selected from the eastern region, 9.9% from central and 29.9% from the western region of China, which was far more than the recommended sample size of at least 10 subjects per item of the original motivation scale [30].

### Indicators and variables

The dependent variable of this study is the primary health workers’ performances in delivering public health services, for which we used the proxy indicator “the proportion of their working time spent on public health activities in the past month”. In the questionnaire, the health workers were asked about the items of public health activities they were engaged with, including health education, health promotion, management of lifestyles, the guidance of behaviors, etc. At the same time, they were asked to fill the proportion of time they spent on each item of public health work they were engaged with. The key independent variable of this study is the intrinsic working motivation level of health workers. This indicator was measured in a 15-item work motivation scale developed based on Self-Determination Theory and relevant empirical researches [23, 31], which covers and assesses five dimensions of work motivation with 3 questions pertaining to the dimension of intrinsic motivation (e.g. “Because I enjoy doing what I do at work every day”) [30]. The Chinese version of this work motivation scale has been measured and verified for its reliability and validity [23]. The level of intrinsic work motivation was measured by three items of scale: i) my working motivation originates from that I enjoy what I do every day at work; ii) my working motivation originates from that I enjoy my tasks at work; iii) my working motivation originates from that I find what I do pretty interesting. Every item was assigned 0–10 scores with a higher score representing greater importance of the item to the respondents. The intrinsic motivation level could be indicated by the mean score of the three items because the items indicate the intrinsic motivation factor with similar strength [30].

In addition to the key independent variable, some other necessary control variables were also included in this study, including the type of PHIs (urban CHCs or rural THCs), whether health workers participated in the family doctor team, external supports for public health work, and health workers’ recognition about the relative importance of curative and public health services. External supports mainly referred to whether information system, performance measurement, and guideline are available to support the integration of public health services into route clinical works. In this study, we measured these external supports through questions on the availability of health record when delivering clinical treatment services, whether income being increased from performance on public health services and the availability of guidelines for the integration of public health services into route work. Health workers’ recognition about curative and public health services was indicated by respondents’ attitudes towards the statement “compared with preventive public health services, clinical services have greater effect on improving population health.” Other individual sociodemographic and work characteristics of health workers included gender (male or female), educational background (high school and below, junior college, bachelor, or master and above), permanent employment status (yes or no), professional title (vice-senior or above, intermediate, junior and others), and license qualification (yes or no).

### Statistical analysis

We firstly conducted descriptive analysis to examine the characteristics of PHIs health workers. Then, a multiple linear regression model at the unit of individual health workers were used to investigate the association between the intrinsic motivation level and behavior of health workers in public health services. The multiple linear regression model is shown as follows:

$${Y}_{i}={\beta }_{0}+\beta {\mathrm{INNER}}_{i}+\delta {X}_{i}+{\varepsilon }_{i}$$

In this regression model, subscript i denotes individual identity. ${Y}_{i}$ is the proportion of working time spent on public health, ranging from 0 to 1. $\beta$ and $\delta$ are the parameters of the econometric model which describe the directions and strengths of the relationship between ${Y}_{i}$ and the factors used to have possible influence on ${Y}_{i}$ in the model. ${\mathrm{INNER}}_{i}$ represents intrinsic working motivation scores of doctors and nurses. ${X}_{i}$ refer to other possibly influencing factors. The error ${\varepsilon }_{i}$ represents unobserved random factors that affect ${Y}_{i}$.

For robustness check, we ran the regression model stratified by different types of institutions among which CHCs represented the urban situation and THCs represented the rural situation, since the healthcare delivery system differs between urban and rural areas of China. In addition to the multiple linear model above, this study also used a generalized linear model with a logit link and the binomial family for robustness check.

The missing values of the dependent variable (the proportion of working time spent on public health services) and the key independent variable (intrinsic motivation) were filled employing multiple imputation method. We filled the missing values of other independent variables with the mean value for continuous variables and the mode value for categorical variables. In all data analysis, the fact that *p*-value < 0.05 was considered statistically significant. All statistical analysis were conducted using Stata, version 17.0 (Stata Corp, College Station, TX, USA).

## Results

### Descriptive results

Table 1 presented the characteristics of doctors and nurses working in PHIs. There were 437 doctors and 366 nurses, a total of 803 health workers being investigated in this study. More than half of them worked in CHCs, and more than 50% of them were females. Most of them have bachelor’s degree or below as their highest degrees, showing relatively low education background of health workers in PHIs. There were 587 doctors and nurses participating in the contracted family doctor team, accounting for 73%. According to the results, 489 (61%) of them had permanent employment status. The majority of doctors (97%) and nurses have got license qualification.

**Table 1:** Basic information of primary care doctors and nurses: n (%)

Characteristics	Doctors	Nurses	Total
Institution			
CHCs	261 (54)	218 (46)	479
THCs	174 (55)	143 (45)	317
Gender			
Male	201 (98)	4 (2)	205
Female	234 (39)	361	595
Highest degree			
Junior college’s degree or below	116 (41)	164 (59)	280
Bachelor’s degree or above	320 (61)	201 (39)	521
Contracted family doctor			
Yes	317 (54)	270 (46)	587
No	74 (46)	87 (54)	161
Permanent employment			
Yes	322 (66)	167 (34)	489
No	90 (32)	189 (68)	279
Professional title			
Senior or above	53 (77)	16 (23)	69
Intermediate	192 (58)	137 (42)	329
Junior	155 (57)	118 (43)	273
Others	35(28)	89 (72)	124
Professional qualification			
Yes	415(53)	361 (47)	776
No	19(83)	4 (17)	23

Table 2 showed the mean percentage of working time spent on public health services. The mean percentage of all primary health workers’ was (38.98 ± 29.82)%. In nurses and doctors, the percentages were (45.64 ± 32.82)% and (32.99 ± 25.43)%, respectively. We can notice that on average, nurses spent more time on public health work than doctors. Mean scores of intrinsic work motivation and relevant three items were presented in Table 3. The mean score of all primary health workers’ intrinsic motivation was 6.59 ± 2.48 (with 10 being the highest level of intrinsic motivation).

**Table 2:** Mean percentage of time spent on public health activities

	N	Mean(%)	St. Err.(%)	t-value	*p*-value
Nurses	331	45.64	32.82	5.75	< 0.001
Doctors	368	32.99	25.43		
Total	699	38.98	29.82

**Table 3:** Mean scores of intrinsic working motivation items

	Total		Nurses		Doctors	
	N	Mean score	N	Mean score	N	Mean score
Im1-I enjoy doing what I do at work every day	764	6.59 ± 2.63	366	6.68 ± 2.64	398	6.49 ± 2.62
Im2- I enjoy my tasks at work	762	6.4 ± 2.67	364	6.59 ± 2.59	398	6.22 ± 2.73
Im3- The work I do is pretty interesting	759	6.77 ± 2.60	361	6.67 ± 2.61	398	6.85 ± 2.59
Intrinsic motivation	758	6.59 ± 2.48	361	6.65 ± 2.50	397	6.53 ± 2.47

### Correlation between intrinsic motivation and performances on public health services

In the multiple linear regression model, the influencing factors of percentage of time on public health services were examined, and the results were shown in Table 4. It was revealed that, with other variables being controlled, higher intrinsic work motivation was related to a lower proportion of time input on delivering public health services (β: -1.01, *p* < 0.05). The result also indicated that doctors had a lower probability of spending more time on public health than nurses (β: -13.57, *p* < 0.01). Health workers in the urban CHCs were more likely to spend more time on public health activities than those in rural THCs (β: 14.20, *p* < 0.01).

**Table 4:** Multiple linear regression analysis

	β	St. Err	*p*-value	[95% CI]	
**Intrinsic motivation**	-1.01^**^	0.41	0.01	-1.82	-0.20
**Institute**	14.20^***^	2.24	0.00	9.81	18.59
**Worker**	-13.57^***^	2.42	0.00	-18.32	-8.83
**Gender**	-2.72	2.32	0.24	-7.27	1.82
**Contract team**	17.27^***^	2.55	0.00	12.28	22.27
**Monthly income**	-0.21	0.28	0.45	-0.08	0.03
**Permanent employment**	9.40^***^	2.19	0.00	5.10	13.70
**License qualification**	2.57	5.98	0.67	-9.18	14.31
**Highest degree**					
Master and above	Ref				
Bachelor	4.80	3.17	0.13	-1.43	11.03
Junior college	5.62	3.79	0.14	-1.83	13.07
High school and below	12.59^**^	5.66	0.03	1.48	23.69
**Professional title**					
Vice-senior and above	Ref				
Intermediate	-2.88	3.73	0.44	-10.19	4.43
Junior and below	-0.73	3.97	0.85	-8.52	7.06
**Clinical-prevention recognition**^**#**^					
1	Ref				
2	-7.51	3.93	0.06	-15.23	0.21
3	-6.96	3.90	0.07	-14.61	0.68
4	-11.06^***^	4.19	0.00	-19.29	-2.84
**Exterior support**	5.04^***^	1.76	0.00	1.59	8.49
**Constant**	12.18	10.28	0.24	-8.00	32.35

*N* = *803, R-squared* = *0.228, F* = *15.903*

^***^ p < *0.01*

^**^ *p* < *0.05*

^*#*^* “Clinical-prevention recognition” refers to respondents’ attitudes toward the statement “Compared with preventive public health services, clinical services have a greater effect on improving population health”. 1* = *totally disagree, 2* = *disagree, 3* = *agree, 4* = *totally agree*

Whether or not participating in the contracted family doctor team might also be associated with the input on public health services. Those in the contract team spent relatively more time on public health services than those who were not (β: 17.27, *p* < 0.01). In addition, doctors and nurses who were permanent staff had on average 9.40% more public health time than those without the permanent employee status (β: 9.40, *p* < 0.01). No significant difference was observed in the percentage of public health activity time among health workers with different levels of professional titles. Whether or not having qualifications was not correlated with their time input on public health services either.

Primary health workers’ perceptions on the relative importance of curative services and public health services to health improvement were also associated with their behaviors in public health services. Those who held the strongest perceptions that preventive care had a greater effect on improving population health than curative care were likely to spend more time on public health activities (β: -11.06, *p* < 0.01). Another significant influencing factor was external support. The availability of more external supports for delivering public health services was positively associated with health workers’ time working on public health services (β: 5.04, *p* < 0.01).

#### Robustness check results

A generalized linear model (GLM) was utilized to check the robustness of the results in the multiple linear model. The results of GLM also showed that higher intrinsic work motivation was negatively related to the percentage of time spent on public health services as the marginal effect of intrinsic motivation on the proportion of time was -0.05 (*p* = 0.155). This means that with other variables held constant, 1 score higher in intrinsic motivation might indicate 5% reduction of the time primary health workers put on public health services, with similar correlation direction to the result in the multiple linear model.

After stratifying by the institutions of CHCs and THCs, the significant relationship between intrinsic motivation and the time put on public health services was only observed in CHCs (β: -1.65, *p* < 0.01). The results in the THCs stratification also displayed the negative association between the intrinsic motivation and input on public health services, but the association was insignificant (β: -0.29, *p* = 0.66).

## Discussion

This study explored the correlation between the intrinsic work motivation of primary health workers and their performance in delivering public health services. The analysis found that a higher intrinsic motivation level among doctors and nurses in PHIs was related to a lower proportion of time input on public health services. According to the stratification analysis, this negative association was more significant in the urban CHCs health workers than in the rural THCs group.

In theory, a higher intrinsic motivation level should be associated with more input on their work, seeming to contradict the findings in this study. According to the literature review, health workers with higher intrinsic motivation levels might be internally inspired to input more time and energy on their work, which has been proven as the booster of good performance and sustainability of efforts on work [24, 25]. However, as the questions in the work motivation scale did not differentiate the enthusiasm for preventive services or curative clinical services, the negative relationship between inner working enthusiasm and time input on public health works may be due to that doctors and nurses perceived curative clinical services, instead of preventive services, as the main sources of their work responsibilities and recognition on the career. In other words, stronger intrinsic motivation indicated that they are more inclined to deliver curative services because this type of service could arouse their interest and bring them more sense of achievement. One study [32] also clarified that one of the main components of doctors’ intrinsic motivation is the challenges from clinical medicine towards their intellectual curiosity. Because of the different features of preventive and curative services, compared with public health works targeted at the population level and aimed at preventing diseases, health workers could feel more direct feedback on their contributions to patients’ health from treating and curing diseases. Evidence also showed that doctors paid more attention to the outcomes of curative services [7], and lacked awareness of the importance of preventive services and public health [33]. Therefore, high intrinsic motivation might indicate health workers’ more recognition of curative services, leading to less time on public health services, as was observed and displayed in this study.

The different significance level between the urban CHCs group and rural THCs group was found. In urban CHCs, better support environments, the higher salary level and better career development path [34] could attract more health workers with higher education level and higher professional title level, while those health workers in higher professional position usually have stronger tendency and preference on clinical work compared with preventive health work. For rural THCs, health workers have the limited opportunities to provide clinical services due to stronger attraction of urban hospitals for patients, at the same time they faced relatively higher workload of pubic health works, all of which gradually strengthens their career recognition on public health works and weaken their pursue and motivation to develop clinical work techniques.

Generally, the tendency of health workers to disease-oriented curative services was severe in primary healthcare settings in China, mainly resulting from the following characteristics of Chinese health system. Firstly, the government provided limited financial subsidies for PHIs, and PHIs had to collect revenues for institution operations by themselves. Meanwhile, the earnings from basic public health services were strictly related to the number of people covered [35], with limited space for revenue increase. Consequently, health workers in PHIs were driven to increase their revenue by delivering more clinical treatment services. Secondly, doctors in PHIs only took charge of the delivery of disease treatment services as the public health department was separately set in PHIs, and the package of public health services were usually undertaken by the specialised public health workers in the public health department [36]. Despite the advocation of integrating curative and preventive services, it was hard to change the perception and behaviors of health workers in the short term. Thirdly, clinical medicine and public health were set as two separate majors in medical education in China. Doctors or nurses in PHIs mainly had clinical medicine training background and had limited knowledge and recognition of public health services. Fourthly, under current promotion criteria for health workers in PHIs, their expertise in clinical treatment can directly bring more opportunities to be promoted to higher professional titles or higher levels in hospitals. Lastly, the current public health services package delivered by PHIs in China includes large amount of work on information documenting and repeated procedures [37]. With the poor integration of information systems between clinical and public health services, documenting works related to public health services lead to a high workload and labor input, which would suppress their enthusiasm for delivering public health services.

The emphasis on clinical treatment services and the neglect of public health services can also be reflected in existing research on work motivation. The majority mainly focused on the influencing factors of work motivation among primary health workers. Others studies on the consequences of intrinsic motivation mostly chose either the general work performance as the outcome [27], or short-term clinical indicators such as hours spent in surgery, frequency of patient contacts, quality of clerical works, and the number of hours spent writing reports as the measures of performances [38]. In the meantime, outcomes and indicators concerning public health services, a specific and essential part of healthcare services were ignored.

Some other factors were also correlated to the time input on public health activities. More comprehensive exterior support was an influencing factor positively related to more input on public health works. Other studies also confirmed that information provision, guidelines formulation and economic incentives, also led to more provision of public health services because it can equip doctors and nurses with the necessary guidance and encourage them to participate in public health services [5, 39]. Results also implied that nurses spent relatively more time on it than doctors, which may be attributed to the limited number of health workers in PHIs. Nurses in PHIs were assigned more work on public health services to ensure that doctors could have enough time for clinical treatment services. Studies in other countries also found that compared to doctors, consultation time was longer in nurses. The frequency of attending follow-up visits was higher for nurses, implying nurses’ more working on communication with patients [40]. The analysis also found that doctors and nurses in contracted family doctor teams who were required to keep close contact with community members delivered more preventive services in disease management. In other words, stable relationships and length of communication with patients could both facilitate more provision of preventive and health management services.

Integrating public health services into primary health care is vital to tackling the challenge of chronic disease. However, this study found that in Chinese PHIs, doctors and nurses only input 38.98% of their time on preventive and health management care. Furthermore, from the results, we deduced that health workers, especially those in urban CHCs, still perceived clinical curative services as their main responsibility and major internal driver for exerting work efforts. To inspire intrinsic interests of doctors and nurses in PHIs on public health services, transfer more of their attention to public health services, and keep the intensity and persistence of their efforts on public health services, systematic and comprehensive measures could be proposed based on findings of this study. At the organization level, the performance-based salary method could increase the proportion of income linked with doctors’ and nurses’ performances in improving the health status of patients they served. In this way, they could be motivated to deliver more health management care and preventive services, other than excessive treatment services. The PHIs could use the opportunity of the family doctor system promoted in China to design contracted family doctor services packages to merge the public health services and clinical services. Relevant guidelines could also be designed and provided to facilitate health workers’ practice. At system level, comprehensive efforts from the government and administration departments can be exerted to reduce the workload burden of public health services on health workers. The local government should continue the efforts to increase the financial support for PHIs. The health information system should be improved to strengthen the integration of medical information system and public health system. In addition, the evaluation methods of public health services could transfer from procedure indicators to more outcome indicators.

This study has several limitations. First, the observational nature of our study limited our ability to draw any causal inference from our findings. The results can only explain the association between intrinsic work motivation and behavior in public health services and found some hints to improve the motivation of health workers in PHIs on public health services. Secondly, the measurements of time input on public health services were based on self-developed questions. They were only used as a proxy indicator of the performance of health workers on public health services, and therefore caused insufficient objectivity and representativeness. Lastly, analysis in this study was based on self-reported data, which may have the risk of bias from the health workers’ memory.

## Conclusion

In this study, it was found that doctors and nurses working in Chinese primary health institutions input limited working time on public health services. Their intrinsic work motivation was found to have a negative association with the time input on public health services, and the association was more significant in community health centers of urban areas. The measurement of intrinsic motivation and findings of this study revealed that health workers in PHIs in China perceived clinical disease treatment services as their primary responsibility and their internal drivers of working and tended to neglect public health services. System-level supportive and organisation-level incentive measures should be constantly improved and implemented to motivate health workers in PHIs to integrate public health services into their routine clinical work.

## Data Availability

The datasets analysed during the current study are not publicly available due to the confidentiality of some information but are available from the corresponding author on reasonable request.
